# The Role of Mycorrhizal Fungi in the Inter and Intraspecific Competition of *Nicotiana glauca* and *Vachellia gerrardii*

**DOI:** 10.3390/plants14060858

**Published:** 2025-03-10

**Authors:** Abdelmalik M. Adam, Thobayet S. Alshahrani, Abdulaziz A. Alqarawi, Basharat A. Dar, Jahangir A. Malik, Ahmed M. Abd-ElGawad

**Affiliations:** Department of Plant Production, Faculty of Food & Agriculture Sciences, King Saud University, P.O. Box 2460, Riyadh 11451, Saudi Arabia; aalqarawi@ksu.edu.sa (A.A.A.); bdar@ksu.edu.sa (B.A.D.); jmalik@ksu.edu.sa (J.A.M.)

**Keywords:** root parameters, photosynthesis, competition, relative yield, aggressivity

## Abstract

A competition experiment between *Vachellia gerrardii* and invasive *Nicotiana glauca* Graham was conducted to assess the impact of Arbuscular Mycorrhizal Fungi (AMF) symbiosis on the inter and intraspecific competition between the two species. Seedlings were established under mono and mixed plantations with different species proportions (3:1, 2:2, 1:3) and plant densities (1, 2, 3, and 4 plants/pot) for mixed and mono planting respectively, with and without AMF. The vegetative growth parameters (height, leaf area and number, total dry weight/plant, relative yield, relative yield total), roots characteristics (length, surface area, volume, tips number), competitive interaction (aggressivity), and physiological traits (chlorophyll *a*, chlorophyll *b*, photosynthesis, stomatal conductance) were measured to evaluate plant responses to AMF symbiosis and competition. The results revealed that AMF symbiosis significantly enhanced the vegetative parameters (leaf area, height, and total dry weight) in both species under mono and mixed plantations compared to plants without AMF. Under AMF treatment, in the interspecific competition, most vegetative and root parameters of *N. glauca* were higher than *V. gerrardii*. At inoculant and species proportions, the relative yield of *N. glauca* exceeded that for *V. gerrardii*; however, *N. glauca* was more aggressive towards *V. gerrardii*. *N. glauca* root indices were higher than *V. gerrardii* under inter and intraspecific competition. Simultaneously, for both species, in monoculture plantations, most parameters decreased as plant density increased, wherein the decrease was higher for plants grown without AMF. Photosynthesis increased in AMF treatment, particularly for *N. glauca*. In conclusion, AMF promoted the growth of invasive *N. glauca* more than native *V. gerrardii,* particularly in terms of the root system. Our results provide a critical perspective that the AMF has the potential to contribute and facilitate the invasion of *N. glauca,* as well as support it with a competitive advantage over *V. gerrardii*, thus highlighting its potential role in shaping plant–plant interaction in invaded habitats.

## 1. Introduction

Plant invasion usually results in a clear reduction in plant biodiversity and community stability, leading to an impact on indigenous plant species [1,2]. In addition, invasive plants can change soil physiochemical properties through competition and interactions with soil microbial communities [3]. Therefore, competition is considered one of the key processes determining plant community structure and dynamics [4,5], which is often arbitrated by nutrients and water availability [6]. Competition is caused due to the limited availability of resources such as water or nutraingredients to any individual plant during its growth. Intraspecific competition occurs between individuals of the same species, and the intensity of competition depends on the level of the limiting resource. However, interspecific competition depends on the species density [7]. In general, many studies indicate that intraspecific competition in plants is stronger than interspecific competition due to similar needs for resources [8]. But, in some cases (competition between native and invasive plants), interspecific competition becomes more intense than intraspecific competition [9]. In general, studies indicate that competition reduces the ability of plants to acquire their essential elements for growth, and thus the degree of intra and interspecific competition can change the competitive balance between invasive and native plants [10].

*Vachellia gerrardii* and invasive *Nicotiana glauca* Graham plants are found in several parts of the southwestern region of Saudi Arabia, such as Khamees Mushait, Al-Baha, Al-Mandag and Al-Karrar [11,12]. *N. glauca*, as an invasive species, is widely considered one of the most destructive plants, resulting in ecological problems worldwide. This plant has been increasing in terms of both population and area, dominating and competing with the natural habitats of native wild plants [13,14]. *N. glauca* has invaded large areas of the Southwestern region of Saudi Arabia [15] and became a dangerous plant in that region over the last few years [12]. *N. glauca* is among the numerous species that have the potential to damage biodiversity or alter ecosystems [13,16]. The presence of toxic substances in the different parts of this plant is believed to contribute to its allelopathic impacts, allowing it to dominate over native plant species [17].

Plant canopies and root areas are overlapped by other plants and living organisms, which creates competitive interaction and environmental stress that challenges their survival [6]. Interactions between plants, soil, and microbes play an important role in determining the structure of plant communities [18]. Moreover, in some cases, complex feedback loops may help plants become more invasive and competitive [19]. The success of invasive plant species is usually related to their competitiveness [20]. Different competitive interactions can impact plant biomass production and facilitate the invasion process [21]. Scientists have proposed that the competitive advantages of native and invasive plants mainly depend on their relation with associated microorganisms [22]. Soil microorganisms act as one of the key drivers in different plant interactions and their community structures. Such interactions not only affect the local species but also endanger the whole ecosystem as long as invasive plants have a mutualistic relationship with native soil microorganisms, e.g., Arbuscular Mycorrhizal Fungi (AMF), where invasive plants receive benefits from AMF symbiosis rather than native plants [23]. AMF can provide several benefits to the host plant such as increased access to water and important nutrients like nitrogen, phosphorus, potassium, carbon, and other micronutrients [24]. AMF interaction might play an important and direct role in shifting the balance of competition between invasive and native plant communities [25]. The competitive dynamics among plants can be altered by the presence of AMF, which plays a crucial role in improving plant nutrition and enhancing their ability to tolerate stress. Different types of plants can either enhance or suppress the AMF association of neighbouring plants, thereby impacting the results of plant interactions [26]. In addition, invasive plants greatly affect the richness and diversity of AMF [27], whereas invasive plants that are not dependent on AMF or have less dependence inhibit mycorrhiza, thereby reducing the competitive advantage of native mycorrhizal plants [28]. Invasive plants that depend on AMF have a selective effect on the formation of the fungal community by promoting their preferred symbiotic species and inhibiting other, less preferred fungi [29]. The preference of the invasive plant for more efficient AMF may alter the richness and abundance of other symbiotic mycorrhizal fungi, and their composition thus facilitate the growth of the invasive plant and reduce the performance of the native plant species [28]. Although not all invasive species cause environmental changes, some can selectively disrupt microbial communities to gain an advantage over native competitors [30]. Furthermore, invasive plants may accumulate pathogens that are more harmful to competitors’ plants [31].

Accordingly, this study attempts to assess the role of AMF in the inter and intra-specific competition of *V. gerrardii* and invasive *N. glauca* and their interaction in various planting densities in greenhouse conditions. Furthermore, the study aims to offer a critical perspective on the potential role of AMF in facilitating the invasion of *N. glauca* by providing it with a competitive edge over *V. gerrardii*.

## 2. Results

### 2.1. Effects of Plant Species Proportion on the AMF Colonization Rate (In the Root) and Spore Number (In the Rhizosphere) of V. gerrardii and Invasive N. glauca Seedlings Grown in Mixed Plantations

Table 1 shows the infection rates of AMF in the roots of *V. gerrardii* and *N. glauca* seedlings grown in mixed planting. The results revealed that species proportion did not show a significant effect on the AMF (mycelium; *p* = 0.5, arbuscular; *p* = 0.11, vesicular; *p* = 0.15) infection rates of *V. gerrardii* and *N. glauca* seedlings. Also, no significant difference was found in the AMF infection rate between the two plant species grown in mixed planting, where *p* values were 0.18, 0.15, and 0.65 for the mycelium, arbuscular, and vesicular, respectively.

**Figure 1:**
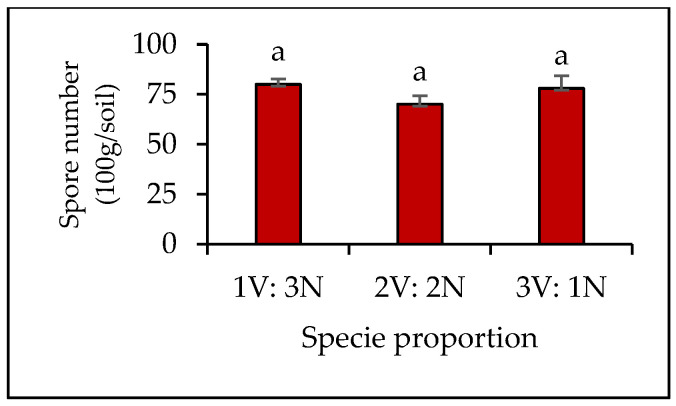
AMF spore number for soils of *V. gerrardii* and *N. glauca* grown in mixed plantations with different species proportion. Note: V = *V. gerrardii*; N = *N. glauca.* Similar letters above bars indicate no significant difference at *p* < 0.05 (LSD test).

In the soils of the mixed plantations of *V. gerrardii* and *N. glauca*, no significant differences were observed in the abundance of AMF spores between treatments of plant species proportion (*p* = 0.34). The average spore number was 80, 70, and 78 spores/100 g of soil in the species proportions 1 *V. gerrardii* (V):3 *N. glauca* (N), 2 *V. gerrardii*:2 *N. glauca*, and 3 *V. gerrardii*:1 *N. glauca*, respectively (Figure 1).

### 2.2. Effect of AMF and Species Proportion/Plant Density on Vegetative Part of V. gerrardii and N. glauca Grown in Mixed Plantations

**Figure 2:**
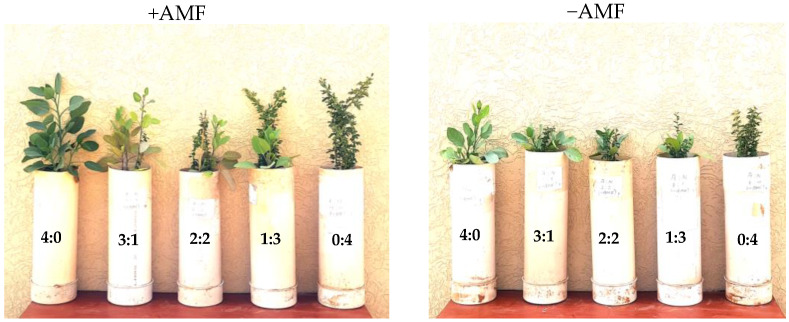
Effect of AMF and species proportion on vegetative growth of *V. gerrardii* and *N. glauca* seedlings established in mixed plantations. Species proportion of *N. glauca* to *V. gerrardii*.

The height of *V. gerrardii* increased in AMF inoculum treatment and increased with a decrease in the proportion of *N. glauca* seedlings (Table 2) (Figure 2). On the other hand, *N. glauca* showed a slight reduction in height with increasing species proportion of *V. gerrardii* (3 *V. gerrardii*:1 *N. glauca*). Under the non-AMF-treated seedlings, species proportion did not significantly affect the heights of *V. gerrardii* and *N. glauca* seedlings (Table 2).

The leaf number of both species was significantly affected by AMF inoculant (*p* = 0.0001), species (*p* = 0.0001), and species proportion (*p* = 0.0002) (Table 2). The number of leaves of the *V. gerrardii* seedlings decreased as the density of *N. glauca* increased in the presence of AMF. However, the number of leaves on *N. glauca* remained constant across all mixed plantations in the presence of AMF (Table 2). The number of leaves of *V. gerrardii* decreased with increasing levels of *N. glauca* in the absence of AMF treatment. Regarding *N. glauca* in the non-AMF treatment, seedlings showed an increase in leaf number as its proportion increased (Table 2).

*V. gerrardii* seedlings treated with AMF had a greater leaf area at all plant densities compared to non-treated seedlings. Seedlings with a higher proportion of species had a greater leaf area compared to seedlings with a lower proportion, irrespective of the inoculant treatment. For *N. glauca*, seedlings treated with AMF exhibited a noticeable increase in leaf area compared to seedlings without AMF at all proportions. Seedlings with lower species proportion had a higher leaf area compared to seedlings with a higher proportion at both inoculant levels. This means that the increase in the number of *N. glauca* had led to intraspecific competition among the seedlings; nevertheless, the intraspecific competition in the AMF treatment was lower than in seedlings without AMF (Table 2) (Figure 2).

### 2.3. Effect of AMF and Species Proportion/Plant Density on Total Dry Weight of V. gerrardii and Invasive N. glauca Grown in Mixed Plantations

**Figure 3:**
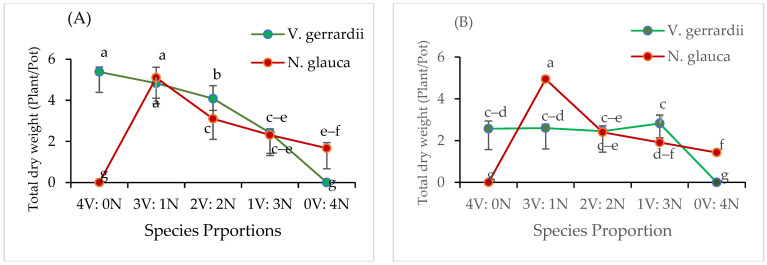
Replacement diagram displaying dry weight for *V. gerrardii* and *N. glauca* in a replacement series with AMF inoculant (**A**) and without AMF inoculant (**B**). Note: V = *V. gerrardii*; N = *N. glauca.* Different letters above graphs indicate a significant difference at *p* < 0.05 (LSD test).

In mixed plantations, total dry weight per seedling was significantly affected by AMF inoculant (*p* < 0.0001), species (*p* = 0.002), plant proportion (*p* < 0.0001), and the interaction between AMF inoculant and species (*p* = 0.0007) (Figure 3). The average total dry weight per plant for *V. gerrardii* in the AMF treatment decreased with an increasing proportion of *N. glauca* (Figure 3A). *V. gerrardii* and *N. glauca* seedlings had dry weights of 4.08 g and 3.1 g, respectively, at a ratio of 2:2 plants (0.50:0.50). Under the non-AMF treatment conditions, *N. glauca* seedlings exhibited a slight decrease in dry weights as the species proportions increased in the pot. In contrast, *V. gerrardii* seedlings maintained consistent dry biomass levels regardless of species proportions (Figure 3B). However, in the species proportion (0.75 A:0.25 N), the average total dry weights were 4.94 g for *N. glauca* and 2.6 g for *V. gerrardii*, indicating that *N. glauca* was affected more with intraspecific competition.

### 2.4. Effect of AMF on Relative Yield and Relative Yield Total of V. gerrardii and N. glauca Seedlings

Relative yield was significantly affected by plant species (*p* = 0.0002) and the species proportion treatment (*p* < 0.0001) (Table 3). The increase in species proportion of *N. glauca* resulted in a decrease in the relative yield of *V. gerrardii* in the AMF treatment. In *N. glauca*, the relative yield also decreased as its proportion increased. The relative yield of *N. glauca* was consistently higher than that of *V. gerrardii* across all species proportions. Relative yield totals in AMF treatment were higher than in the non-AMF treatment. The relative yield of *V. gerrardii* showed a decrease in the non-AMF treatment as the proportion of *N. glauca* increased, particularly in the 1 *V. gerrardii*:3 *N. glauca*. However, the decrease is lower in comparison to that in AMF-treated seedlings of the same species ratio. (Table 3).

### 2.5. Aggressivity Index of V. gerrardii and N. glauca Grown with and Without AMF Inoculant

The effect of the AMF inoculant on the aggressiveness of the two species was not significant (*p* = 0.78), implying a similar response from both species. However, species proportion had a significant effect on species aggressivity (*p* = 0.004) (Table 4). The aggressivity index of the AMF treatment reveals that *N. glauca* displays a higher level of aggression towards *V. gerrardii*. Nevertheless, as the proportion of *N. glauca* in the mixture increases to 2:2 plants, its aggressivity decreases. *N. glauca* displayed increased aggressiveness towards *V. gerrardii* in the absence of AMF treatment, particularly in the ratio of 3 *V. gerrardii*:1 *N. glauca*. *N. glauca* demonstrated a high level of aggression towards *V. gerrardii*, regardless of the presence or absence of AMF. However, the presence of AMF resulted in a reduction in aggression, except for the proportion 1 *V. gerrardii*:3 *N. glauca* (Table 4).

### 2.6. Effect of AMF and Plant Proportion/Density on the Root Parameters of V. gerrardii and N. glauca Seedlings Grown in Mixed Plantations

**Figure 4:**
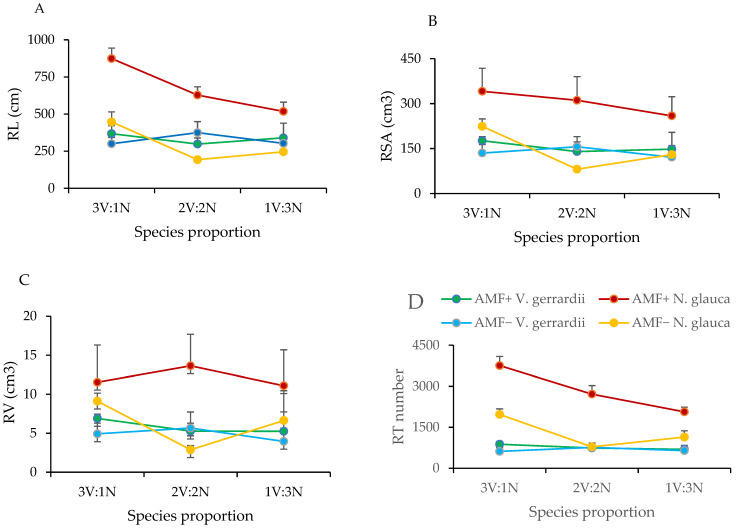
Total RL (**A**), RSA (**B**), RV (**C**), and RT number (**D**) for *V. gerrardii* and *N. glauca* grown in mix-culture in different species proportions with and without AMF. V = *V. gerrardii;* N = *N. glauca*.

The total root length in the diverse plantation was significantly influenced by plant species (*p* = 0.0001), AMF inoculant (*p* < 0.0001), species proportion (*p* = 0.005), the interaction between AMF inoculant and species (*p* < 0.0001), and the interaction between species and proportion (*p* = 0.001). The AMF-treated seedlings of *V. gerrardii* in the mixed plantations displayed a slight augmentation in root length at the species proportions of 3 *V. gerrardii*:1 *N. glauca* and 1 *V. gerrardii*:3 *N. glauca*, in comparison to their non-AMF-treated counterparts. However, in the 2 *V. gerrardii*:2 *N. glauca* ratio, seedlings that were not inoculated with AMF exhibited a longer root length compared to the seedlings that received AMF treatment. The total root length of *N. glauca* seedlings showed a marked increase when treated with AMF as opposed to those that were not treated, across all proportions (Figure 4A).

The total root surface area was significantly impacted by plant species (*p* = 0.004), AMF inoculant (*p* = 0.0016), and the interaction between plant species and AMF (*p* = 0.0098). In *V. gerrardii*, the application of AMF resulted in an increase in the root surface area in the treated seedlings. However, this increase was not significantly different from the non-AMF-treated seedlings across all species proportions. On the other hand, in *N. glauca*, the seedlings experienced significant advantages from AMF compared to the non-AMF-treated seedlings, especially in the proportion of 3 *V. gerrardii*:1 *N. glauca* (Figure 4B).

Root volume was notably influenced by species (*p* = 0.03), whereas the presence of AMF inoculant (*p* = 0.05) and species proportion did not have a significant effect (*p* = 0.95). The root volume of *V. gerrardii* seedlings showed an increase after being inoculated with AMF, regardless of the species proportion, although this difference was not statistically significant when compared to the non-AMF-treated seedlings (Figure 4C). In the case of *N. glauca* seedlings, the application of AMF led to a noticeable increase in root volume when compared to its counterpart and *V. gerrardii* seedlings grown with and without AMF inoculant. This suggests that *N. glauca* derives greater advantages from AMF association than *V. gerrardii*, as shown in Figure 4C.

Root tip numbers were significantly affected by AMF (*p* < 0.0001), plant species (*p* < 0.0001), species proportion (*p* = 0.0003), and the interaction between species and AMF inoculant (*p* < 0.0001). Despite being inoculated with AMF, the root tips in *V. gerrardii* seedlings did not show a considerable increase along the entire species proportion. However, *N. glauca* showed the highest root tip numbers in the AMF-treated seedlings compared to the non-AMF-treated plants (Figure 4D).

### 2.7. Effect of AMF and Plant Proportion on the Chlorophyll a and b Levels of V. gerrardii and N. glauca Seedlings Grown in Mixed Plantations

**Figure 5:**
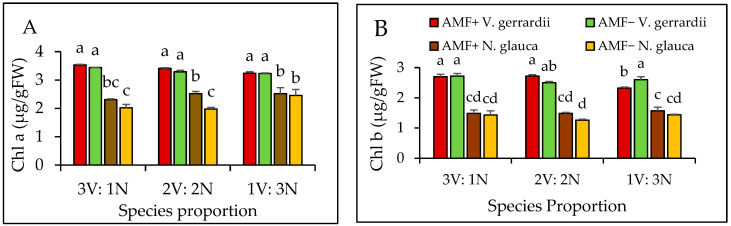
Chlorophyll *a* (**A**) and chlorophyll *b* (**B**) for *V. gerrardii* and *N. glauca* grown in mixed plantations. Note: V = *V. gerrardii;* N = *N. glauca.* Different letters above bars indicate a significant difference at *p* < 0.05 (LSD test).

Plant species in the mixed plantation had a significant impact on chlorophyll *a* content (*p* = 0.0001). Furthermore, chlorophyll *a* content was influenced by AMF (*p* = 0.01) and the proportion of species (*p* = 0.008) (Figure 5A). The chlorophyll *a* content of *V. gerrardii* seedlings treated with AMF displayed a slight increase throughout proportions compared to the non-AMF-treated seedlings, although the difference was not statistically significant. In contrast, the application of AMF to *N. glauca* seedlings resulted in a significant elevation in chlorophyll *a* content. This effect was observed across all species proportions, indicating the positive impact of AMF treatment. Notably, *V. gerrardii* displayed higher levels of chlorophyll *a* content in both AMF inoculant levels compared to *N. glauca* plants (Figure 5A).

Plant species had a significant impact on the content of chlorophyll *b* (*p* = 0.0001), while no significant effects were observed for AMF inoculant (*p* = 0.33) and species proportion (*p* = 0.07); however, their interactions (*p* = 0.056) were marginally affected chlorophyll *b*. *V. gerrardii* seedlings treated with AMF in all proportions showed a slight increase in chlorophyll *b* content compared to those without AMF, but the difference was not statistically significant (Figure 5B). In contrast, *N. glauca* seedlings had higher levels of chlorophyll *b* content in the AMF-treated group across all species proportions, yet the difference was not significantly different from the seedlings without AMF in the same proportions (Figure 5B).

### 2.8. Effect of AMF and Plant Proportions on the Photosynthesis and Stomatal Conductance of V. gerrardii and N. glauca Seedlings Grown in Mixed Plantations

The photosynthesis rate in the mixed plantations was found to be significantly influenced by various treatments. These include species (*p* = 0.004), AMF inoculant (*p* = 0.00001), and plant proportion (*p* < 0.0001) (Table 5). In the case of *V. gerrardii* seedlings, AMF treatment resulted in significantly higher photosynthesis rates compared to the seedlings that were not inoculated with AMF, even when considering the same species proportion. Furthermore, as the proportion of *V. gerrardii* decreased in the pot, the photosynthesis rate also decreased, primarily due to interspecific competition. However, the seedlings treated with AMF maintained the highest photosynthesis values in comparison to their non-treated counterparts. The highest photosynthesis rate was observed in *N. glauca* seedlings that were treated with AMF at a species ratio of 3 *V. gerrardii*:1 *N. glauca*. A decrease in the photosynthesis rate was noted with an increase in the proportion of *N. glauca* (Table 5).

Stomatal conductance in the mixed plantations was significantly influenced by plant species (*p* = 0.0004), species proportion (*p* < 0.0001), and their interaction (*p* = 0.02). In the case of *V. gerrardii* seedlings, the highest stomatal conductance value was observed in the presence of AMF seedlings. As the proportion of *V. gerrardii* per pot decreased, the stomatal conductance decreased in both AMF-treated and non-AMF-treated seedlings. Nevertheless, the seedlings treated with AMF exhibited a higher stomatal conductance compared to the non-AMF-treated seedlings (Table 5). Stomatal conductance in *N. glauca* was significantly higher in the species proportion 3 *V. gerrardii*:1 *N. glauca* when treated with AMF, as indicated by the recorded values. Additionally, an increase in the proportion of *N. glauca* per pot led to a noticeable decrease in stomatal conductance, especially in seedlings without AMF (Table 5).

**Figure 6:**
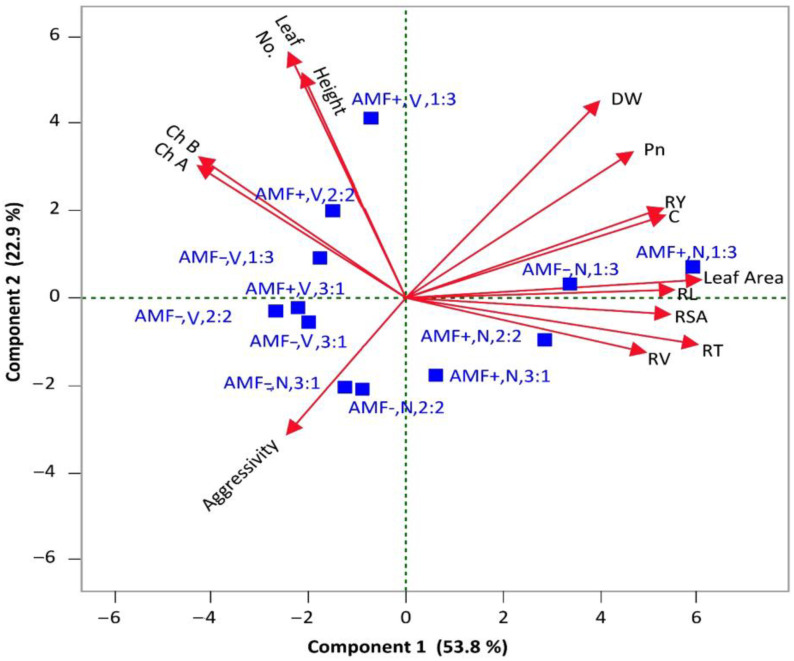
PCA for parameters of *V. gerraradii* and *N. glauca* seedlings established at mixed plantations with and without AMF. AMF+, V, 1:3 = *V. gerrardii* seedlings treated with AMF at species proportions of 1:3, 2:2, and 3:1 *V. gerrardii*: *N. glauca*; AMF−, V, 1:3 = *V. gerrardii* seedlings without AMF at species proportions of 1:3, 2:2, and 3:1 *V. gerrardii*:*N. glauca*; AMF+, N, 1:3= *N. glauca* seedlings treated with AMF at species proportions of 1:3, 2:2, and 3:1 *V. gerrardii*: *N. glauca*; AMF−, N, 1:3 = *N. glauca* seedlings without AMF at species proportions of 1:3, 2:2, and 3:1 *V. gerrardii*: *N. glauca*.

Figure 6 illustrates that the first two principal components (PCs) on the X and Y axes accounted for a total of 76.7% variance, with PC1 and PC2 showing a variance of 53.8% and 22.9%, respectively. Our results in principle component analysis (PCA) indicated that the root length (RL), relative yield (RY), and stomatal conductance (C) of *N. glauca* are negatively correlated to aggressivity at a proportion of 1:3 with and without AMF treatment. In the lower left quadrant, root volume (RV) and root tip number (RT) are highly correlated under the AMF treatment at a 1:3 plant/pot proportion. Additionally, RL and leaf area were positively correlated under both AMF and non-AMF treatments at a species proportion of 1:3 plant/pot in the same quadrant. Similarly, chlorophyll *a* and chlorophyll *b* showed a positive correlation under AMF at species 2 *V. gerrardii*:2 *N. glauca* in the upper right quadrant, while height and leaf number are closely related in AMF treatment at 1 *V. gerrardii*:3 *N. glauca* plant/pot.

### 2.9. Effects of Plant Densities on the AMF Colonization Rate and Spore Number of V. gerrardii and Invasive N. glauca Seedlings Grown in Mono Plantations

In the monoculture plantations of *V. gerrardii*, plant density has a marginal effect on the infection rate of arbuscular (*p* = 0.05) and did not affect mycelium (*p* = 0.11) and vesicular mycorrhiza (*p* = 0.24). Arbuscular mycorrhiza had a higher value, being recorded in the density 1 plant/pot. However, the lowest infection rates were recorded by mycelium (at 1, and 2 plant densities) and arbuscular (at 3 and 4 plant densities) (Table 6).

For *N. glauca*, plant densities did not impact the infection rates of mycelium (*p* = 0.86), arbuscular (*p* = 0.84), and vesicular (*p* = 0.67) mycorrhiza. Nevertheless, arbuscular mycorrhiza showed the highest infection rate along all plant densities. However, the lowest infection rates were recorded for vesical along all plant densities (Table 6).

**Figure 7:**
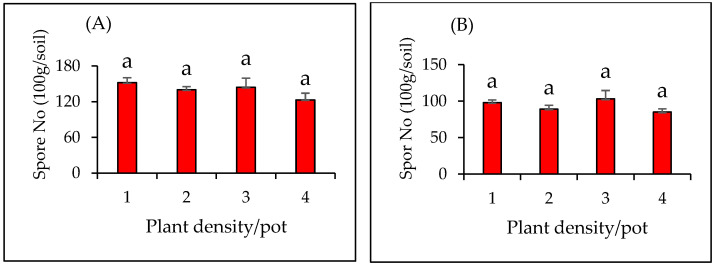
AMF spore in soils of (**A**) *V. gerrardii* and (**B**) *N. glauca* seedlings grown in mono plantings with different plant densities. Similar letters above bars indicate no significant difference at *p* < 0.05 (LSD test).

Also, no significant differences were found in the spore numbers between plant densities for both plant species established in mono plantings (*p* = 0.58 and 0.31 for *V. gerrardii* and *N. glauca* respectively). AMF spores in *V. gerrardii* soils were 152, 140, 144, and 123 for plant densities 1, 2, 3, and 4 respectively. However, AMF spores in *N. glauca* soils were 98, 89, 103, and 85 at densities 1, 2, 3, and 4, respectively (Figure 7A, B).

### 2.10. Effect of AMF and Plant Density on Height, Leaf Number, and Leaf Area of V. gerrardii and N. glauca Grown in Mono Plantations

The height of *V. gerrardii* in monoculture plantations was significantly influenced by both AMF inoculant (*p* = 0.0001) and plant density (*p* = 0.03). In the seedlings treated with AMF inoculant, height exhibited an increase as the density decreased. The height of plants grown without AMF experienced a significant decrease as plant density increased (Table 7; Figure S1). 

The height of *N. glauca* at monoculture plantations was significantly affected by AMF inoculant (*p* = 0.0001). Increasing plant density led to a reduction in the height of the seedlings, irrespective of whether they were AMF or non-AMF seedlings. Seedlings grown without AMF experienced a 35.18% decrease in height at a density of four plants per pot, while those with AMF showed a slightly lower reduction of 32.66% (Table 7; Figure S2).

The leaf number of *V. gerrardii* was significantly affected by AMF inoculant (*p* = 0.005) and plant density (*p* = 0.0001). Seedlings with AMF showed the highest values of leaf number in all plant densities compared to the non-AMF-treated seedlings (Table 7). A reduction in leaf number was observed with an increase in plant density for both treated and non-treated seedlings. However, the presence of AMF resulted in a higher leaf number compared to the untreated seedlings (Table 7).

The number of leaves on *N. glauca* seedlings in monoculture was significantly influenced by the AMF inoculant (*p* = 0.0007), while density (*p* = 0.057) and the interaction between AMF and density did not have a significant effect on leaf numbers (*p* = 0.63). Seedlings treated with AMF exhibited a remarkable increase in leaf number when contrasted with untreated seedlings (Table 7).

The monoculture plantations displayed significant variations in leaf area per plant for *V. gerrardii* seedlings under AMF treatment and the chosen plant density (*p* = 0.0002, *p* = 0.001), respectively (Table 7). The increase in plant densities resulted in a decline in leaf area, regardless of whether the plants were associated with AMF or not. The presence of AMF alleviated the influence of plant density on leaf area, contrasting with the non-AMF-treated seedlings (Table 7).

Leaf area in *N. glauca* was significantly impacted by plant density and the presence of AMF (*p* < 0.0001) (Table 7). Seedlings with AMF treatment exhibited higher leaf area compared to the seedlings without AMF in the same density. The increase in plant density resulted in a decrease in leaf area for both AMF and non-AMF-treated seedlings; however, seedlings inoculated with AMF exhibited increased leaf area when compared to those without AMF (Table 7). 

### 2.11. Effect of AMF and Plant Density on Total Dry Weight of V. gerrardii and Invasive N. glauca Grown in Mono Plantations

**Figure 8:**
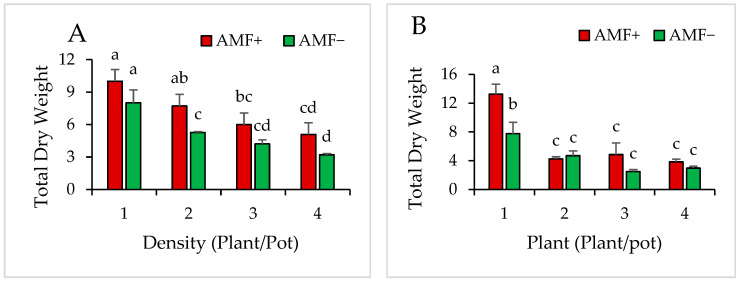
Total dry weight for *V. gerrardii* (**A**) and *N. glauca* (**B**) grown in monoculture in different densities with and without AMF. Different letters above bars indicate a significant difference at *p* < 0.05 (LSD test).

In the context of monoculture planting, findings demonstrated significant impacts for AMF (*p* = 0.003) and plant density (0.00001) on the total plant dry weight of *V. gerrradii* seedlings. In both AMF and non-AMF-treated seedlings, dry weight decreased progressively as plant density increased. Nevertheless, the decline in dry weight was more moderate in AMF-treated seedlings than in non-inoculated seedlings at the corresponding density (Figure 8A).

The total dry weight of *N. glauca* cultivated in monoculture was found to be significantly influenced by the AMF inoculant (*p* = 0.006), density (*p* < 0.0001), and the interaction between the two factors (*p* = 0.026). The presence of AMF effectively reduced the intraspecific competition among *N. glauca* seedlings caused by the higher plant densities. In the case of non-AMF (four plants per pot) seedlings, the average dry weight was measured at 2.98 g. However, the AMF-treated seedlings at the same density displayed a significantly higher average dry weight of 3.83 g. This indicates that *N. glauca* seedlings derived benefits from the AMF inoculant (Figure 8B).

### 2.12. Effect of AMF and Plant Densities on the Chlorophyll a and b of V. gerrardii Seedling Grown in Monoculture Plantations

**Figure 9:**
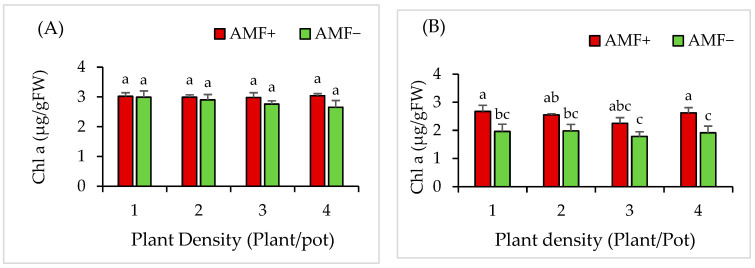
Chlorophyll *a* (**A**) and *b* (**B**) for *V. gerrardii* grown in monoculture plantations. Note: Different letters above bars indicate a significant difference at *p* < 0.05 (LSD test).

In monoculture plantations, the content of chlorophyll *a* in *V. gerrardii* seedlings was not significantly influenced by the AMF inoculant (*p* = 0.12), plant densities (*p* = 0.75), or their interaction (*p* = 0.67) (Figure 9A).

For chlorophyll *b*, AMF had a significant impact on the content of chlorophyll *b* (*p* = 0.0004) in *V. gerrardii* seedlings, whereas plant density did not show a significant effect on chlorophyll *b* (*p* = 0.49). Furthermore, the interaction between the AMF inoculant and plant density did not show a significant effect on chlorophyll *b* content (*p* = 0.92 (Figure 9B)).

### 2.13. Effect of AMF and Plant Densities on the Chlorophyll a and b of N. glauca Seedling Grown in Monoculture Plantations

**Figure 10:**
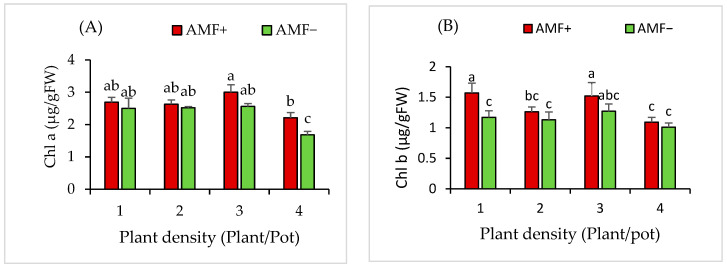
Chlorophyll *a* (**A**) and *b* (**B**) for *N. glauca* seedlings grown in monoculture plantation. Note: Different letters above bars indicate a significant difference at *p* < 0.05 (LSD test).

The chlorophyll *a* content in *N. glauca* monoculture plantations was significantly influenced by the AMF (*p* = 0.01) and plant densities (*p* = 0.0006), while their interaction did not yield a significant effect (*p* = 0.601). The AMF treatment had a profound impact on the levels of chlorophyll *a*. Notably, the seedlings treated with AMF exhibited significantly higher values of chlorophyll *a* when compared to their counterparts at the same density level (Figure 10A).

The chlorophyll *b* of *N. glauca* seedlings grown in monoculture plantation were significantly impacted by the AMF treatment, as demonstrated (*p* = 0.0006). The density of plants did not show a statistically significant effect on chlorophyll *b* levels in *N. glauca* seedlings (*p* = 0.49). AMF-treated seedlings exhibited the highest chlorophyll *b* content across all plant densities, in contrast to the untreated seedlings. Specifically, the density of one plant per pot resulted in the highest chlorophyll *b* content for AMF-treated seedlings, while the lowest value was observed in non-AMF-treated seedlings at a plant density of four plants per pot (Figure 10B).

### 2.14. Effect of AMF and Plant Densities on the Photosynthesis and Stomatal Conductance of V. gerrardii and N. glauca Seedling Grown in Monoculture Plantations

The photosynthesis rate of *V. gerrardii* seedlings grown in monoculture plantations was significantly influenced by AMF inoculant (*p* = 0.04). Conversely, no significant impact was detected for the plant density (*p* = 0.11) or its interaction with AMF (*p* = 0.49). The photosynthesis rate of *V. gerrardii* seedlings was found to be higher with AMF treated seedlings, as opposed to the seedlings without AMF, despite having a similar plant density (Table 8).

The stomatal conductance of *V. gerrardii* seedlings was significantly influenced by AMF treatment (*p* = 0.01). In contrast, the plant density (*p* = 0.10) did not show any significant effects (*p* = 0.9). Stomatal conductance was observed to be higher in the AMF treatment as compared to the non-AMF treatment within each plant density. It was observed that as the plant density increased, there was a decrease in stomatal conductance. However, this decrease was not significantly different and was more pronounced in the non-AMF-treated seedlings compared to the seedlings with AMF (Table 8).

*N. glauca* plants exhibited significant differences in photosynthesis rate as a result of the AMF inoculant (*p* = 0.0001) and plant density (*p* = 0.002), with no significant impact observed for the interaction between plant density and AMF inoculant (*p* = 0.49). AMF significantly improved the rate of photosynthesis in all seedling densities when compared to seedlings that did not receive AMF treatment (Table 8). As the density of plants increased, there was a significant reduction in the photosynthesis rate, particularly observed in seedlings lacking AMF (Table 8).

Stomatal conductance in monoculture plantations of *N. glauca* seedlings was not significantly affected by AMF inoculant (*p* = 0.60). However, plant density had a significant effect (*p* = 0.02) on stomatal conductance. Despite the lack of a significant impact on stomatal conductance in *N. glauca*, the presence of AMF inoculant resulted in higher stomatal conductance in treated seedlings compared to those without AMF. Notably, an increase in plant density per pot was observed to decrease stomatal conductance in the seedlings (Table 8).

**Figure 11:**
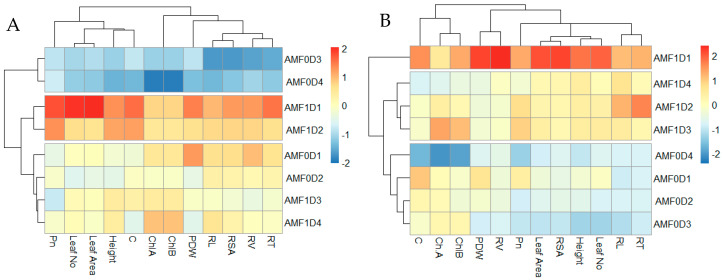
Heatmap for the parameters (root tips number RT; root volume RV; root surface area RSA; plant dry weight PDW; chlorophyll *a* ChA; chlorophyll *b* ChB; stomatal conductance C; photosynthesis rate P) of *V. gerrardii* (**A**) and *N. glauca* (**B**) grown in mono plantings. AMF1D1, AMF1D2, AMF1D3, AMF1D4 = seedlings treated with AMF at plant densities 1−4; AMF0D1, AMF0D2, AMF0D3, AMF0D4 = seedlings without AMF at plant densities 1−4.

For the mono plantings of *V. gerrardii*, heatmap analysis revealed three distinct clusters, which included seedlings without AMF for the plants with densities of 3 and 4 (blue color), seedlings with AMF at densities of 1 and 2 plants (red color), and three seedlings with AMF at plant densities of 3 and 4 and seedlings without AMF at plant densities of 1 and 2. Most of the measured traits showed a positive correlation with AMF treatments at all plant densities, where correlation increases as plant density decreases. However, in untreated plants, the studied parameters showed a negative correlation, particularly at plant densities of 3 and 4 (Figure 11A).

For *N. glauca* plantings, most parameters correlated positively (red color) with AMF treatment at the different plant densities, particularly at 1 plant/pot. The positive correlation decreases with the increase in plant density. On the other hand, seedlings without AMF showed lower values in the most measured traits with no or negative (blue color) correlation, particularly in the higher plant densities. In general, seedlings with AMF treatments showed a positive correlation in studied parameters, while seedlings without AMF correlated negatively with the most studied traits (Figure 11B).

## 3. Discussions

### 3.1. AMF Spores and Colonization Rate in the Roots of V. gerrardii and N. glauca Grown in the Mono and Mixed Plantations

In this study, both plant species (*V. gerrardii* and invasive *N. glauca*) attracted AMF and made a symbiosis relationship with it, with no statistical variation in AMF infection% and spore numbers. The consistency of the AMF colonization rate between *V. gerrardii* and *N. glauca* could be attributed to the ability of both species to form a symbiotic relationship with AMF, and both species were cultivated in a uniform environment. In this regard, the authors of [32] have indicated that *N. glauca* plants have the ability to form associations with AMF in various environments. Also, *V. gerrardii* has been reported for its ability to establish symbiotic relationships with AMF. According to [33], AMF colonization was observed in the roots of *V. gerrardii* across different environments in Saudi Arabia. Furthermore, a competition study conducted by the authors of [34] revealed that the rate of AMF colonization was similar between invasive and native plants in numerous cases. Also, several studies have indicated that the abundance of AMF spores is influenced by the host plant species as well as other factors such as soil pH and moisture content [35]. Conversely, several studies have reported either a decrease [28] or an increase [36] in AMF spores between co-concurrent invasive and native plants growing in invaded soils, as reported in [37].

### 3.2. Responses of Vegetative Parameters of V. gerrardii and N. glauca Grown in Mixed and Monoculture Plantations to AMF and Competition

The performance of *V. gerrardii* and *N. glauca* in the greenhouse condition showed different responses to the AMF inoculant levels, which indicates that AMF inoculant plays an important role in the interference and interactions between the two species. Our results demonstrated that *N. glauca* has great capability of competition (due to high total yield) and maintains a dominant growth pattern in both conditions (with and without AMF). These findings follow the results of the authors of [38], who stated that the total yield of invasive and native plants can increase with AMF colonization. This could be attributed to the stronger mutualistic relationship between invasive species and AMF compared to native species, resulting in a high competitive advantage for invasive plants [39]. Interestingly, the highest level of aggression was observed when the plant proportion was 3 *V. gerrardii*:1 *N. glauca*, highlighting the ability of *N. glauca* to dominate in various environmental settings. In mixed plantations, it was observed that *V. gerrardii* experienced a higher impact from the interspecific competition, while *N. glauca* was more influenced by intraspecific competition, as evidenced by the dry weight production and vegetative parameters per plant. In *N. glauca*, it is also observed that the population density is effectively controlled by the intense intraspecific competition that occurs within the species. In this regard, studies explained that non-native plants produce extensively (double to four times) higher biomass and showed more aggressive growth than that of indigenous plants, suggesting their better ability to exploit accessible resources [40]. The ability of plant competition for the available resources in the soil is an important issue because competition can determine their growth, reproduction, and biomass. According to the findings reported by [41], competition between *Proteus vulgaris* and invasive *Ipomoea purpurea* was greatly impacted by AMF.

In our study, the interspecific competition between seedlings was extensive in reducing relative yield (RY) for *V. gerrardii* when the two species are grown together, suggesting that *N. glauca* gains more RY and advantages from the AMF inoculant. These findings are in line with the results of a competition study between invasive and native plants, which stated that AMF can change the competitive relationship between native and invasive plants [42]. Moreover, the AMF role is linked with the inter and intraspecific competition between plant species [43]. In a study conducted by the authors of [41], *Iopmoea purpurea* weed showed positive AMF growth responses in mono and mixed plantation, and its growth biomass in the mixed planting was significantly superior to those in monocultures, demonstrating that the growth of *I. purpurea* under the mixed situations was better improved by AMF compared to the growth of the *Proteus vulgaris* plain crop. Regardless of the supportive role of AMF in increasing the competitiveness of invasive *N. glauca* over *V. gerrardii*, this superiority can also be linked and attributed to the allelopathic substances that may be exuded by invasive *N. glauca* roots. Scientists have reported that the invasive plant may compete with native plant species by releasing allelochemical compounds [44] and growing rapidly, thereby suppressing neighbouring native plants [45]. This can be a crucial mechanism, allowing them to invade and colonise new environments [46]. Therefore, the allelochemical compounds released from invasive plants may provide the species with a competitive advantage against native plant species [47].

The outcomes of the effect of AMF and species proportion on vegetative parameters in mixed and mono plantations resulted in different patterns of interspecific and intraspecific competition between the two plants. For example, in the mixed plantations, *N. glauca* had a higher leaf area at all proportions regardless of AMF compared to *V. gerrardii,* thereby exerting stronger interspecific competition against *V. gerrardii*. This could be attributed to the morphological variations between the two species. In this context, the authors of [48] stated that differences in morphological structure are linked to biomass allocation, which is considered important for plant development, helping them to acquire resources during competition. Additionally, our findings align with the research conducted by the authors of [39], who reported a reduction in the shoot biomass of indigenous plants due to competition, while simultaneously enhancing the shoot biomass of invasive plant species. The competitive character of invasive plants that benefit from their symbiotic relation with AMF may also be ascribed to improved N and P uptake, which has already been facilitated by AMF hyphae [49]. Many studies have indicated that invasive plants have greater advantages in many different trait groups such as fast growth rate, vigour, size, leaf area, and shoot allocation than native plants [50]. It is well known that AMF increases the ability of plants to absorb water and nutrients. Therefore, the suppression of AMF inoculants reduces the competitive ability and vigour status of native plant species [51].

### 3.3. Responses of Root Parameters of V. gerrardii and N. glauca Growing in Mixed and Monoculture Plantation to AMF and Competition

The presence of AMF in mixed plantations had a favourable effect on the growth and development of *N. glauca* in interspecific competition, particularly in terms of root traits. Our study confirmed that *N. glauca* exhibited increased root parameters compared to *V. gerrardii* under the influence of AMF throughout the entire species proportion. Conversely, the root parameters of native *V. gerrardii* did not demonstrate the same degree of benefits as *N. glauca*. This result is consistent with the findings documented in [52], which demonstrated that, in the mixed plantations of the invasive *Rhus typhina* and the native *Acer truncatum*, root competition from the invasive species significantly reduced the space available for the root system of *A. truncatum*. As a result, this led to more pronounced detrimental effects on the growth of root parameters in the native *A. truncatum*. Furthermore, [42] revealed that, in the presence of the interspecific competition between invasive and native plants, AMF influenced root parameters such as length, surface area, volume, and tips in both plant species, and, in most cases, invasive plants derived greater advantages compared to native plants.

Scientists reported that the low growth of root systems in plants directly limits the plant’s nutrient and resource assimilation [53]. Therefore, the native *V. gerrardii*, probably absorbed little amounts of nutrients when competing with the invasive *N. glauca*. As a result, the exploitation of soil resources by *V. gerrardii* roots decreased more prominently, which may indirectly have resulted in a competitive advantage for *N. glauca* in resource utilisation. This is in line with the fact that non-native invaders are known to influence nutrient fluxes, availability, and their uptake [54], which can change the physiology and growth rate of native species, as well as alter the abundance of species within plant communities [55].

In this study, the invasive species *N. glauca* shows a greater ability to outcompete the native species *V. gerrardii* in utilising soil resources through its increased root and symbiotic relationships with AMF. This symbiosis further enhances the invasive potential of *N. glauca*. Our findings are in agreement with results reported by the authors of [52], who stated that invasive *R. typhina* attracted more AMF when competing with the native *A. truncatum*. Also, AMF decreased the root network of the invasive *R. typhina* compared to the native *A. truncatum*. Moreover, invasive plants can produce allelochemical compounds that inhibit neighbouring native plants via the disruption of beneficial belowground microbial mutualisms, or altered soil resources [56]. Allelopathy could have a large impact on native species across the globe [57]. Allelopathy and resource competition are considered to be the second primary mechanisms responsible for the loss of biodiversity in forest ecosystems [58]. Allelopathy was reported to be more important than resource competition in mediating the reduction in plant biodiversity [59]. A study conducted by the authors of [60] stated that the root traits of the introduced invasive species *Chromolaena odorata* populations were significantly different from those of the native populations. Many results have confirmed the role of AMF in increasing the roots of invasive plants when grown singly or mixed with native plant species [38]. The differences in the roots and morphological traits between invaders and native plants can impose interspecific competition advantages for invaders rather than the native neighbours [61]. The fine root demonstrates that the plant root system has a better ability to uptake soil resources (nutrients, water) [62]. This may suggest the competitive advantages of invasive *N. glauca* over the native *V. gerrardii*.

In this study, the presence of AMF in monoculture plantations significantly increased root system traits, such as root length, root surface area, root volume, and root tip, for both plant species across varied densities when compared with their counterparts. This suggests that the invasive *N. glauca* exhibits a higher capacity to attract AMF through its fine root structure compared to the native *V. gerrardii*. According to the authors of [63], AMF inoculant increases the length of root hyphae, which offers help in soil nutrient absorption and, therefore, encourages plant growth [64]. The growth of root parameters in invasive species, such as *Baptisia alba*, and the native species *Petrophile biternate*, has been significantly enhanced by the application of AMF inoculant in monoculture plantation, as reported by several researchers. This improvement can be attributed to the facilitation provided by AMF, as highlighted in the study conducted by the authors of [43].

### 3.4. Responses of Physiological Parameters of V. gerrardii and N. glauca Grown in Mixed and Monoculture to AMF and Competition

The differences in chlorophyll *a* and chlorophyll *b* content in plants under AMF inoculant have been described in different plant species [65]. Studies have proposed that the use of AMF may lead to better plant biomass, plant water content, as well as other vegetative parameters, in addition to allowing the enhanced quality and quantity of chlorophyll contents [66]. In our study, the chlorophyll content among seedlings in mixed plantations was found to be consistent regardless of the presence of AMF or species proportion. However, variations in chlorophyll content were observed between the two plant species. Despite the lack of a significant effect on chlorophyll content from the presence of AMF in *N. glauca* within mixed plantations, seedlings treated with AMF demonstrated increased levels of chlorophyll *a* and chlorophyll *b* compared to their untreated counterparts. On the other hand, *V. gerrardii* had superior chlorophyll *a* and chlorophyll *b* compared to *N. glauca*. The results from a study on *Z. mays* showed that the plant showed increased chlorophyll content when inoculated with AMF [65]. On the other hand, a reduction in the total chlorophyll content was reported in *Thymus daenensis* and *T. vulgaris* plants when inoculated with two different types of AMF (*Rhizophagus intraradices* and *Funneliformis mosseaea*) [67]. In general, the differences in the content of plant chlorophyll are considered an important aspect that indirectly identifies the plant’s photosynthesis capacity [68].

AMF might enhance the photosynthetic assimilation in plants by enhancing their various vegetative parameters, finally resulting in the improvement of photosynthesis efficiency [69]. This finding is in accordance with our results, which revealed that the AMF inoculant showed a slight increase in the photosynthesis rate, particularly for invasive *N. glauca* compared to *V. gerrardii* in mixed and mono plantations. AMF plants may display enhanced stomatal conductance. Therefore, the plants may have the ability to keep their stomata open for a longer time than non-AMF-treated plants [70].

## 4. Materials and Methods

### 4.1. Plant Materials and Growth Conditions

Newly produced seeds of *V. gerrardii* and *N. glauca* were collected from the AL-Baha region (19°53′56″ N, 41°34′17″ E), located in the southwestern part of Saudi Arabia. Additionally, soil samples under both species were collected from the same site for the purpose of preparing the mycorrhizal inoculum. All the collected materials were taken to the lab of Forestry and Range Science, Faculty of Food and Agriculture Sciences, King Saud University (KSU).

### 4.2. Interspecific Competition Between V. gerrardii and N. glauca

To measure the intensity of competition between *V. gerrardii* and invasive *N. glauca* plants, the seeds of both species were germinated in a mixture, and the total density was 4 plants per tube (16 cm diameter and 50 cm height). The density levels were 3:1, 2:2, and 1:3 (*V. gerrardii*:*N. glauca*). Seedlings were cultivated with (+) and without (–) AMF. Twenty-five grams of inoculum soil containing AMF spores (100 spore/10 g) were added to each AMF treatment. Young seedlings were established for a specific density in experimental tubes (16 cm diameter and 50 cm height), filled with a mixture of soil consisting of sand and silt (3:1) with five replicates per treatment. For the tubes that contain AMF, the soil was inoculated before seedlings were established. The total number of tubes in this experiment was 30, and the experimental design was factorial (3 levels of density* ± AMF×5 replicates) in a Complete Randomised Design (CRD). Each tube received a weekly intake of 250 mL of water, and 250 mL of Hogland’s solution was added every 2 months. The experiment was conducted in a greenhouse (College of Food and Agriculture Sciences, King Saud University (KSU)) and lasted for 7 months.

### 4.3. Intraspecific Competition for V. gerrardii and N. glauca

To measure the intensity of competition, the seedlings of each species (*V. gerrardii* and *N. glauca*) were established in monoculture with (+) and without (–) AMF in the densities of 4, 3, 2, and 1 plant per tube. The seeds of each species were sown separately in the experimental tubes (16 cm diameter and 50 cm height) and filled with a mixture of soil consisting of silt and sand (3:1) with four replicates per treatment. After germinating, seedlings were thinned for a specific density. For the tubes that contain AMF, soils were inoculated before sowing the seeds. Next, 25 g of inoculum soil containing AMF spores (100 spore/10 g) was added in each AMF treatment. Hoagland solution was added to the experiment during the growth period (7 months). This experiment was set as a randomised complete block design with four replicates for every density/species, and tubes were set randomly within blocks in the greenhouse. The experiment consisted of 4 levels of density (for each species) * ± AMF × 4 replicates. In both experiments, all tubes were placed in a greenhouse at 30 °C under natural daylight.

### 4.4. Data Collection

#### 4.4.1. AMF Colonization in Seedlings Root and Spore Extraction

AMF was extracted from the root sample of each treatment for both species using the method described in [71] and modified by that in [72]. The roots of seedlings were cleaned with tap water (4 °C) to remove the attached soil and then cleaned with potassium hydroxide (KOH, 10%) solution and, consequently, staining with trypan blue in lactophenol as stated in [73]. According to this method, roots were cut for segments and checked for AMF occurrence using a light microscope at 400× magnification. The infection rate of AMF (mycelium, vesicles, and arbuscules) in the roots of both species was calculated as stated in the formula below:

(1) $$\%\;\mathrm{Colonization}\;=\frac{\mathrm{Total}\;\mathrm{number}\;\mathrm{of}\;\mathrm{AM}\;\mathrm{positive}\;\mathrm{segments}}{\mathrm{Total}\;\mathrm{number}\;\mathrm{of}\;\mathrm{segment}\;\mathrm{studied}!}\times 100$$

The spores of AMF were extracted from the soil of mono and mixed plantations using the wet sieving and decanting protocol [74]. According to this method, 100 g of soil from each sample was kept in a bucket (1500 mL capacity) containing 1 litre of water. The soil sample was shaken and mixed with water to create soil suspension. The resulting soil suspension was left for 6 min and then sieved using gradual sieves (ASTM-60, ASTM-100, and ASTM-240) for spore extraction. Soil suspension was filtered via Whatman filter paper No. 1. The filter papers that contained AMF spores were examined under an electronic microscope (with 2.5 × 10 magnification) and then spores were counted. Moreover, AMF spores and their species were identified according to their morphological features under the microscope used [74].

#### 4.4.2. Relative Yield (RY)

The relative yields of *V. gerrardii* and *N. glauca* were measured using the formula below, according to the protocol used in [75].

Relative Yield (RY) of *V. gerrardii* = Y_AN_/(pY_AA_) (2)

Relative Yield (RY) of *N. glauca* = Y_NA_/(qY_NN_) (3)

where Y_AN_ indicates the biomass of the *V. gerrardii* seedlings that were established in the mixed planting and p is the primary proportions (75%, 50%, and 25%) of *V. gerrardii* seedlings, duplicated by the Y_AA_, which is the average production of *V. gerrardii* seedlings that was established in monoculture plants (4 plant per tube). Likewise, Y_NA_ indicates the production of *N. glauca* seedlings that was established in mixture with *V. gerrardii* seedlings, and “q” is the amounts of the species (75%, 50%, and 25%), multiplied by the average production of *N. glauca* that was established in monoculture planting (4 plant per pot). The relative yield of the *V. gerrardii* and *N. glauca* seedlings (2 plant species) was used to measure the relative yield total per tube.

#### 4.4.3. Relative Total Yield (RTY)

Measuring RTY is an indicator that shows whether the two plant species participate in resources or interfere with each other by comparing their biomass (per tube) in monoculture plantations to their biomass (per tube) in mixed plantations [76]. The formula below is applied to identify RTY:

RTY = pRY_A_ + qRY_P_ (4)

where p = initial proportion of *V. gerrardii* seedlings in a mixture plant and q is the initial amount of *N. glauca* seedlings in a mixture such that

p + q = 1.(5)

The data of this experiment (replacement series experiments) were analysed following a replacement series diagram. In the case of the competition between the two plant species, each species participates in the total production in the direct ratio according to its proportion in the mixture culture. The yield for each species increases linearly with its proportion in the mixture plants. In the unequal competition, the aggressive plant species will receive more biomass than expected. The aggressivity index for *V. gerrardii* and *N. glauca* seedlings was identified using the formulas below:

Aggressivity of *V. gerrardii* = RY_A_ − RY_N_ (6)

Aggressivity of *N. glauca* = RY_N_ − RY_A_ (7)

#### 4.4.4. Measurement of Chlorophyll (a, b) and Photosynthesis Parameters

To determine chlorophyll content, fresh leaves from *V. gerrardii* and *N. glauca* under each treatment were collected separately. Samples weighing 0.5 g from each treatment were collected in the early morning, brought to the lab directly and weighed by digital balance. The samples were then crushed by mortar and pestle and transferred to test tubes. Each tube was filled with dimethyl formamide (5 mL) and left at room temperature for 24 h. The resulted leaf extracts were filtered via Whatman filter paper No. 1. and placed in a cuvette to measure chlorophyll content using a spectrophotometer (SHIMADZU, Kyoto, Japan, UV1800). Absorbance was read at different wavelengths (664 nm for chlorophyll *a*, and 620 nm for chlorophyll *b*) [77].

The net photosynthesis rate and stomatal conductance were measured using a portable system device (CI-340 handheld photosynthesis system, CID Bio-Science, Camas, WA, USA). The measurements were conducted between 9.00 and 11.00 am in the greenhouse on the healthy leaves of the plant.

#### 4.4.5. Measurements of Above Ground Parameters

The height of both seedling species (*V. gerrardii* and *N. glauca*) was measured separately from soil surface to seedling apex using a 1-m ruler. Also, the leaf area (cm^2^) was measured using a portable leaf area meter (CI-202). Leaf number per seedling was counted and the plant dry biomass (g) was determined after drying the whole plant at 70 °C for 48 h.

#### 4.4.6. Measurements of Bellow Ground System (Root Parameters)

After harvesting, the roots of both plant seedlings were separated from the vegetative part and gently washed with tap water to remove soil particles. The roots were then stained using a red-coloured dye and spread gently over a computer scanner for imaging. The roots were scanned at 600 dpi, and the root images were saved in Tiff format. The root characteristics such as total root length, total root surface area (cm^2^), root volume, and root tip number were analysed using a PC equipped with WinRHIZO version 2004a (Regent Instruments Inc., Québec, QC, Canada) software.

### 4.5. Data Arrangement and Statistical Analysis

All data were subjected to analysis of variance (ANOVA); moreover, upon finding significance, means were separated using a Least Significant Difference (LSD) test at *p* < 0.05. Principal component analysis (PCA) was applied for the data of mixed plantation to capture the maximum variation along X and Y axes, thereby facilitating the interpretation of experimental results. The results were projected in the factor space, and their relationships were interpreted based on the angle and distance from the origin. Additionally, heatmap analysis was performed based on the correlation matrix for mono plantations. Statistical analysis was conducted using Statistix 10.0 software program and JMP Pro.16. Also, the replacement diagram was generated graphically to show species replacement dynamics in mixed plantations.

## 5. Conclusions

Biological invasion is a critical problem facing the world today, leading to damage to ecosystems and biodiversity. The relationship between AMF and plants can affect the competition dynamics between invasive and native plants. In our study, AMF was associated with the changes and growth of *V. gerrardii* and invasive *N. glauca* and enhanced vegetative and root parameters for the two plant species in mono and mixed plantations via the connected AMF hyphae. Meanwhile, the two plant species responded differently to AMF and to the interspecific competition, where the performance of *V. gerrardii* in interspecific competition was lower than *N. glauca*. AMF induced an improvement in several growth parameters including vegetative (leaf area, leaf number, height, plant dry weight, relative yield, relative yield total), physiological (photosynthesis, stomatal conductance), and root traits for *N. glauca* and *V. gerrardii*. The increase in these studied parameters was generally more pronounced and statistically more significant for *N. glauca* than for *V. gerrardii*. Furthermore, *N. glauca* presented with a greater relative yield than *V. gerrardii* under interspecific competition, making the *N. glauca* more aggressive and a stronger competitor than *V. gerrardii*. In conclusion, our results suggest that the invasive *N. glauca* gained higher assistance scores from AMF and more competitive advantage over the native *V. gerrardii*, particularly in the development of the root system. These findings may provide evidence that AMF increases the likelihood of *N. glauca* invasion, giving a perspective with which to recognise the mechanisms of plant biological invasion.

## Figures and Tables

**Table 1:** AMF % in the roots of *V. gerrardii* and *N. glauca* grown in mixed culture with different species proportion.

Species	Plant Species Proportion (*V. gerrardii*:*N. glauca*)	AMF Structural Colonization%		
		Mycelium%	Arbuscular%	Vesicular%
*V. gerrardii*	3:1 (0.75:0.25)	40.0 ± 5.7 a	50.3 ± 5.7 a	53.3 ± 3.3 a
	2:2 (0.50:0.50)	56.6 ± 6.0 a	43.0 ± 3.3 a	45.0 ± 2.8 a
	1:3 (0.25:0.75)	53.3 ± 3.3 a	40.0 ± 5.7 a	71.6 ± 4.4 a
*N. glauca*	3:1 (0.75:0.25)	60.0 ± 10 a	35.0 ± 2.8 a	53.0 ± 3.3 a
	2:2 (0.50:0.50)	50.0 ± 5.7 a	36.6 ± 3.3 a	56.6 ± 3.3 a
	1:3 (0.25:0.75)	61.6 ± 4.4 a	45 ± 5.0 a	54.0 ± 3.4 a
LSD		29.6	21.4	27.8

Note: Same letters within each column represent no significant differences at *p* < 0.05 (LSD test).

**Table 2:** Effect of AMF and species proportion on height, leaf number, and leaf area of *V. gerrardii* and *N. glauca* grown in mixed plantations.

Species	Plant Species Proportion (*V. gerrardii*:*N. glauca*)	AMF Inoculant	Height (cm)	Leaf Number	Leaf Area (cm^3^)
*V. gerrardii*	3:1 (0.75:0.25)	+AMF	37.2 ± 3.86 a	42.0 ± 5.19 a	128 ± 11.47 c
		−AMF	19.8 ± 1.28 bc	21.4 ± 1.60 bc	63 ± 5.49 cd
	2:2 (0.50:0.50)	+AMF	24.6 ± 0.74 b	30.2 ± 0.73 b	88 ± 2.17 cd
		−AMF	18.8 ± 3.52 bc	16.0 ± 2.16 cde	41 ± 5.42 cd
	1:3 (0.25:0.75)	+AMF	17.0 ± 1.44 bc	20.2 ± 1.98 bcd	46 ± 4.34 cd
		−AMF	18.0 ± 2.70 bc	15.8 ± 2.8 cde	33 ± 4.98 d
*N. glauca*	3:1 (0.75:0.25)	+AMF	12.20 ± 1.24 c	10.8 ± 0.73 de	478 ± 34.7 a
		−AMF	12.4 ± 1.50 bc	9.8 ± 0.58 e	215 ± 12.9 b
	2:2 (0.50:0.50)	+AMF	18.4 ± 2.87 bc	11.40 ± 1.02 cde	289 ± 41.3 b
		−AMF	10.8 ± 2.28 c	8.6 ± 0.92 e	124 ± 15.5 c
	1:3 (0.25:0.75)	+AMF	16.4 ± 3.82 bc	10.6 ± 1.36 de	104 ± 14.6 cd
		−AMF	15.2 ± 2.35 bc	10.8 ± 0.80 de	82 ± 5.7 cd
LSD			12.25	10.14	86.51

Note: Different letters within each column represent significant differences at *p* < 0.05 (LSD test).

**Table 3:** Relative yield and relative yield total of *V. gerrardii* and *N. glauca* grown with and without AMF inoculant.

Species	Plant Species Proportion (*V. gerrardii*:*N. glauca*)	AMF Inoculant	Relative Yield (RY)	Relative Yield Total (RYT)
*V. gerrardii*	3:1 (0.75:0.25)	AMF+	3.81 ± 0.29 b	5.29
*N. glauca*		AMF+	5.79 ± 0.7 a	
*V. gerrardii*	2:2 (0.50:0.50)	AMF+	3.21 ± 0.50 bcd	3.235
*N. glauca*		AMF+	3.26 ± 0.43 bcd	
*V. gerrardii*	1:3 (0.25:0.75)	AMF+	1.90 ± 0.14 e	2.035
*N. glauca*		AMF+	2.44 ± 0.32 cde	
*V. gerrardii*	3:1 (0.75:0.25)	AMF−	3.25 ± 0.23 bcd	4.095
*N. glauca*		AMF−	5.46 ± 0.22 a	
*V. gerrardii*	2:2 (0.50:0.50)	AMF−	3.06 ± 0.33 bcd	3.185
*N. glauca*		AMF−	3.31 ± 0.14 bc	
*V. gerrardii*	1:3 (0.25:0.75)	AMF−	2.15 ± 0.60 de	2.27
*N. glauca*		AMF−	2.64 ± 0.31 cde	

Note: Different letters within each column represent significant differences at *p* < 0.05 (LSD test).

**Table 4:** Aggressivity index for *V. gerrardii* and *N. glauca* grown in mixed plantations with and without AMF inoculant.

Plant Species Proportion (*V. gerrardii*:*N. glauca*)	Aggressivety Index	
	+AMF	−AMF
3:1 (0.75:0.25)	−1.98 ± 0.87 bc	−2.21 ± 0.39 c
2:2 (0.50:0.50)	−0.05 ± 0.64 a	−0.24 ± 0.32 a
1:3 (0.25:0.75)	−0.53 ± 0.38 ab	−0.48 ± 0.50 ab

Aggressivity index was identified as (RY of *V. gerrardii*- RY of *N. glauca*). The negative values indicate that the RY of *V. gerrardii* are less than the RY of *N. glauca*; thus, negative values indicate that *N. glauca* is more aggressive than *V. grrardii*. Different letters within each column represent significant differences at *p* < 0.05 (LSD test).

**Table 5:** Effect of species proportion and AMF inoculant on photosynthesis and stomatal conductance for *V. gerrardii* and *N. glauca* in mixed plantation.

Species	Plant Species Proportion *(V. gerrardii:N. glauca*)	AMF Inoculant	Photosynthesis (µmol CO_2_/m^2^/s)	Stomatal Conductance (mol H_2_O/m^2^/s)
*V. gerrardii*	3:1 (0.75:0.25)	AMF+	16.1 ± 0.8 ab	207 ± 25 ab
		AMF−	8.1 ± 1.1 cde	213 ± 19 ab
	2:2 (0.50:0.50)	AMF+	13.5 ± 1.3 abc	198 ± 11 b
		AMF−	6.4 ± 0.4 e	178 ± 9 b
	1:3 (0.25:0.75)	AMF+	9.8 ± 0.8 cde	144 ± 11 b
		AMF−	7.7 ± 1.2 de	151 ± 12 b
*N. glauca*	3:1 (0.75:0.25)	AMF+	18.5 ± 0.9 a	289 ± 22 a
		AMF−	12.7 ± 1.6 bcd	288 ± 25 a
	2:2 (0.50:0.50)	AMF+	13.5 ± 1.3 abc	214 ± 20 ab
		AMF−	10.4 ± 1.3 cde	197 ± 11 b
	1:3 (0.25:0.75)	AMF+	10.6 ± 0.9 cde	167 ± 16 b
		AMF−	7.5 ± 0.6 de	167 ± 12 b
LSD			5.4489	85.3

Different letters within each column represent significant differences at *p* < 0.05 (LSD test).

**Table 6:** AMF infection rates for *V. gerrardii* and *N. glauca* seedlings grown in monoculture plantations with different plant densities.

Plant Density	*V. gerrardii*			*N. glauca*		
	AMF Structural Colonization%			AMF Structural Colonization%		
	Mycelium%	Arbuscular%	Vesicular %	Mycelium%	Arbuscular%	Vesicular%
1	50.0 ± 0.0 a	62.6 ± 9.3 a	63.3 ± 6.6 a	53.3 ± 6.6 a	86.6 ± 3.3 a	48.3 ± 4.4 a
2	41.6 ± 6.0 a	43.3 ± 3.3 ab	45.0 ± 5.0 a	56.6 ± 8.8 a	70.0 ± 11 a	43.3 ± 8.8 a
3	45.0 ± 5.0 a	41.6 ± 7.2 ab	48.3 ± 1.6 a	60.0 ± 10 a	66.6 ± 12 a	46.0 ± 3.3 a
4	33.3 ± 3.3 a	30.0 ± 5.7 b	53.4 ± 8.8 a	46.6 ± 6.6 a	80.0 ± 5.7 a	45.0 ± 2.8 a
LSD	19.2	30.73	27.7	26.6	29.2	17.6

Different letters within each column represent significant differences at *p* < 0.05 (LSD test).

**Table 7:** Effect of AMF and density on height, leaf number, and leaf area of *V. gerrardii* grown in mono plantations.

Plant Density	AMF Inoculant	*V. gerrardii*			*N. glauca*		
		Height (cm)	Leaf Number	Leaf Area (cm^3^)	Height (cm)	Leaf Number	Leaf Area (cm^3^)
1	AMF+ AMF−	50.5 ± 6.5 a	72.2 ± 14.3 a	239 ± 50 a	54.5 ± 12.3 a	23.7 ± 2.9 a	577 ± 59 a
		36.2 ± 2.7 bc	43.7 ± 2.9 bcd	134 ± 12 b	27.0 ± 1.4 bcd	16.7 ± 1.2 bc	321 ± 34 bc
2	AMF+ AMF−	49.0 ± 2.6 a	53.7 ± 3.9 b	164 ± 15 ab	35.7 ± 5.0 ab	18.2 ± 2.5 ab	410 ± 33 ab
		34.0 ± 1.5 bcd	36.2 ± 2.0 bcd	111 ± 10 b	23.0 ± 1.7 bcd	14.2 ± 1.1 bc	189 ± 15 cd
3	AMF+ AMF−	42.2 ± 2.9 ab	45.7 ± 2.2 bc	136 ± 13 b	36.7 ± 9.6 ab	18.7 ± 2.0 b	322 ± 46 bc
		27.7 ± 2.2 cd	29.7 ± 1.6 cd	84 ± 4 b	13.7 ± 1.6 cd	12.5 ± 1.0 bc	136 ± 8 d
4	AMF+ AMF−	42.0 ± 5.4 ab	45.0 ± 6.0 bcd	129 ± 15 b	36.7 ± 7.1 abc	17.2 ± 1.3 bc	231 ± 19 cd
		24.7 ± 2.4 d	28.0 ± 2.9 d	72 ± 9 b	17.5 ± 1.5 d	14.5 ± 1.0 c	124 ± 12 d
LSD		10.79	17.56	98	18.82	5.31	156

Different letters within each column represent significant differences at *p* < 0.05 (LSD test).

**Table 8:** Effect of AMF on photosynthesis and stomatal conductance in *V. gerrardii* and *N. glauca* growing in monoculture plantations.

Plant Density (Plant/Pot)	AMF Inoculant	*V. gerrardii*		*N. gerrardii*	
		Photosynthesis (µmol CO_2_/m^2^/s)	Stomatal Conductance (mol H_2_O/m^2^/s)	Photosynthesis (µmol CO_2_/m^2^/s)	Stomatal Conductance (mol H_2_O/m^2^/s)
1	AMF+	13.8 ± 2.05 a	320.0 ± 67.6 a	18.7 ± 0.9 a	230.0 ± 24.6 a
	AMF−	10.5 ± 0.82 bc	238.0 ± 39.4 abc	14.3 ± 3.1 abc	210.5 ± 28.2 ab
2	AMF+	13.2 ± 0.47 ab	302.7 ± 9.07 ab	16.3 ± 2.4 ab	169.5 ± 25.4 abc
	AMF−	10.9 ± 1.2 abc	248.0 ± 26.9 abc	8.2 ± 1.1 bc	181.7 ± 48.87 abc
3	AMF+	9.9 ± 1.07 c	264.2 ± 19.1 abc	16.9 ± 1.9 ab	168.5 ± 15.4 abc
	AMF−	9.8 ± 1.08 c	213.5 ± 4.83 bc	7.5 ± 1.4 c	168.0 ± 18.7 abc
4	AMF+	10.9 ± 0.35 abc	228.0 ± 18.5 bc	11.1 ± 1.7 abc	146.5 ± 19.5 bc
	AMF−	9.97 ± 0.83 c	188.2 ± 6.04 c	5.9 ± 0.7 c	115.0 ± 7.93 c
LSD		5.2057	145.44	8.70	122.48

Different letters within each column represent significant differences at *p* < 0.05 (LSD test).

## Data Availability

All data used in this study are published in the paper.

## Supplementary Materials

The following supporting information can be downloaded at: https://www.mdpi.com/article/10.3390/plants14060858/s1, Figure S1: Growth of *V. gerrardii* with and without AMF in monoculture plantations in 1 (A), 2 (B), 3 (C), and 4 (D) plants densities.; Figure S2: Growth of *N. glauca* with and without AMF in monoculture plantations in 1 (A), 2 (B), 3 (C), and 4 (D) plants densities.
