# Supplementary material for: The Role of Mycorrhizal Fungi in the Inter and Intraspecific Competition of Nicotiana glauca and Vachellia gerrardii
Source: Plants (Basel). 2025 Mar 10;14(6):858. doi: 10.3390/plants14060858 (PMC11945343; doi:10.3390/plants14060858)
Supplement: Supplementary file 1 [file plants-14-00858-s001.zip › plants-3489395-supplementary.pdf]

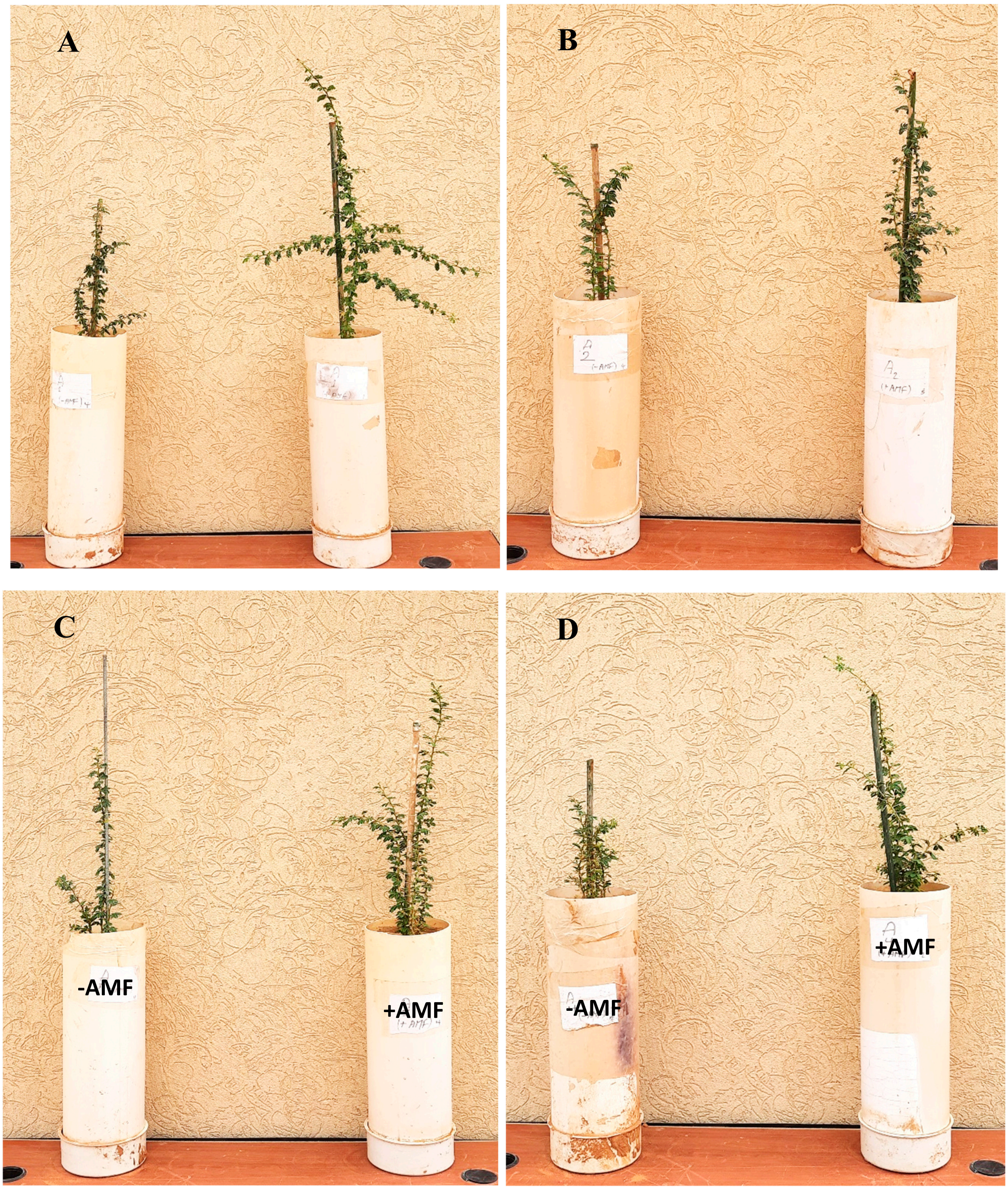

Figure S1. Growth of *A. gerrardii* with and without AMF in monoculture plantations in 1 (A), 2 (B), 3 (C) and 4 (D) plants densities.

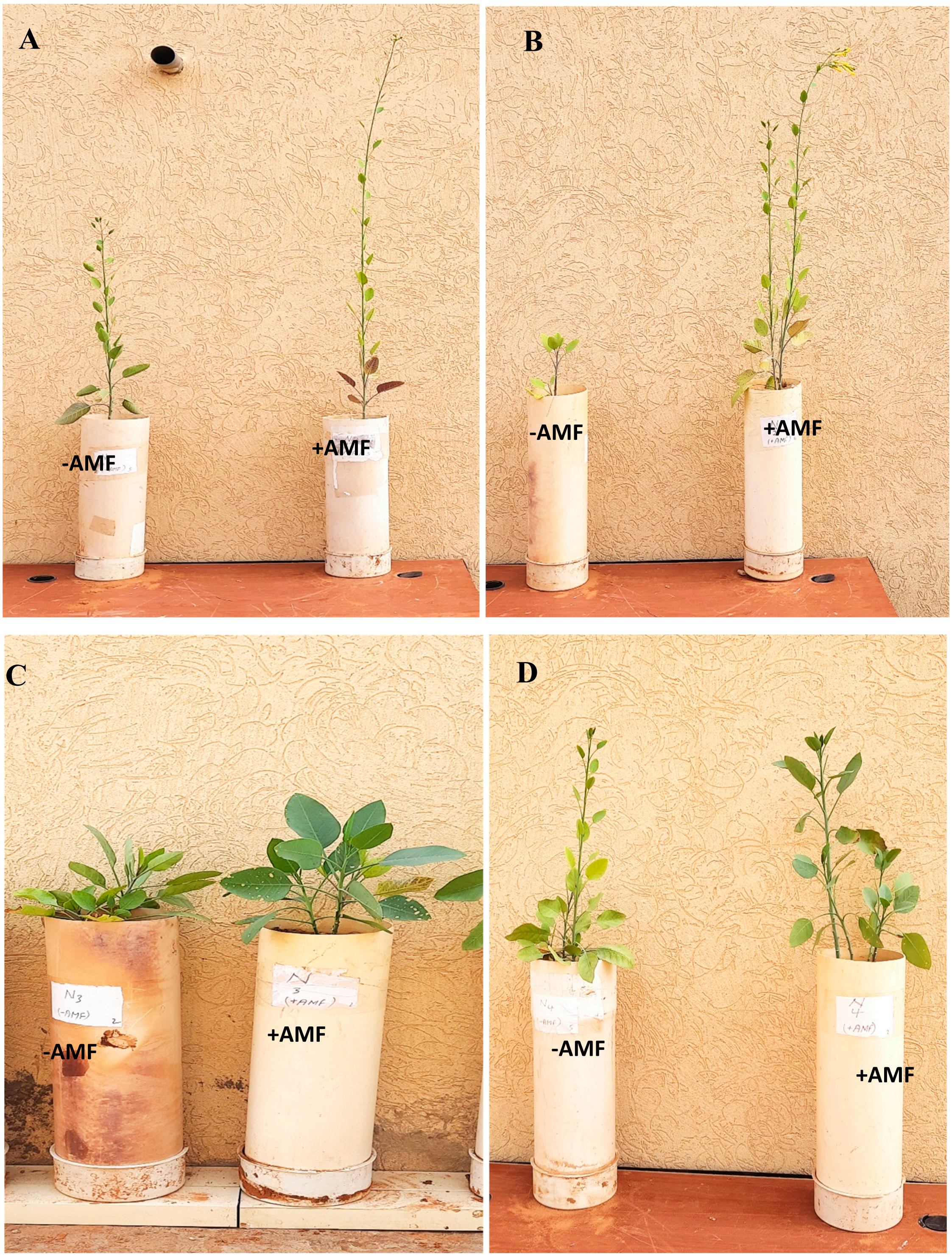

Figure S2. Growth of *N. glauca* with and without AMF in monoculture plantations in 1 (A), 2 (B), 3 (C) and 4 (D) plants densities.
